# Sympathetic Nervous System Activity in Preschoolers Who Stutter

**DOI:** 10.3389/fnhum.2019.00356

**Published:** 2019-10-09

**Authors:** Bridget Walsh, Anne Smith, Sharon L. Christ, Christine Weber

**Affiliations:** ^1^Communicative Sciences and Disorders, Michigan State University, East Lansing, MI, United States; ^2^Speech, Language, and Hearing Sciences, Purdue University, West Lafayette, IN, United States; ^3^Human Development and Family Studies, Purdue University, West Lafayette, IN, United States

**Keywords:** stuttering, children, speech, autonomic nervous system, sympathetic arousal, electrodermal activity, blood pulse volume, pulse rate

## Abstract

**Background:**

In our Dynamic Pathways, account, we hypothesized that childhood stuttering reflects an impairment in speech sensorimotor control that is conditioned by cognitive, linguistic, and emotional factors. The purpose of this study was to investigate potential differences in levels of sympathetic arousal during performance of speech and non-speech tasks between children who do and do not stutter.

**Methods:**

Seventy-two preschool-aged children participated in the study, 47 children who stutter (CWS; 38 boys) and 25 children who do not stutter (CWNS; 18 boys). We recorded skin conductance and blood pulse volume (BPV) signals, indices of sympathetic arousal, during higher/lower load speech tasks (structured sentence production and picture description) and non-speech tasks (jaw wagging and forceful blowing). We included a measure that reflects children’s attitudes about their communication skills and a parent-report assessment of temperament.

**Results:**

We found no significant differences between preschool CWS and CWNS in phasic skin conductance response amplitude or frequency, BPV, and pulse rate for any of the experimental tasks. However, compared to CWNS, CWS had, on average, significantly higher skin conductance levels (SCL), indexing slowly changing tonic sympathetic activity, across both speech and non-speech experimental conditions. We found distinctive task-related profiles of sympathetic arousal in both groups of preschool children. Most children produced the highest levels of sympathetic arousal in the physically demanding blowing task rather than in speech, as seen in previous studies of adults. We did not find differences in temperament between the two groups of preschool children nor a relationship among behavioral indices of temperament and communication attitude and physiological measures of sympathetic arousal.

**Conclusion:**

We did not find that atypically high, speech-related sympathetic arousal is a significant factor in early childhood stuttering. Rather, CWS had higher, on average, task-related tonic SCLs across speech and non-speech tasks. A relationship among behavioral measures of temperament and physiological measures of sympathetic arousal was not confirmed. Key questions for future experiments are how the typical coupling of sympathetic and speech sensorimotor systems develops over childhood and adolescence and whether task related developmental profiles follow a different course in children who continue to stutter.

## Introduction

Many theorists have concluded that experimental and clinical evidence related to the onset and development of stuttering in childhood points to the central roles played by speech motor, language, and emotional factors (Walden et al., 2012; Ambrose et al., 2015; Smith and Weber, 2017). Experimental evidence supports the critical roles of speech sensorimotor systems and the mediating effects of language processes in early childhood stuttering (Silverman and Ratner, 2002; Anderson et al., 2005; MacPherson and Smith, 2013; Weber-Fox et al., 2013; Spencer and Weber-Fox, 2014; Walsh et al., 2015; Kreidler et al., 2017; Usler et al., 2017). Our understanding of how emotional factors may affect speech production in stuttering, especially in early childhood, is far more limited. One approach used to explore “emotional” factors in stuttering is to record physiological signals that index autonomic nervous system (ANS) functions. Whether patterns of increased or decreased ANS activation map onto specific cognitive states is a matter of open debate (Kreibig, 2010). It is widely acknowledged, however, that ANS activity reflects diverse behavioral processes, and increased sympathetic nervous system (SNS) activity accompanies increases in cognitive and physical effort and reflects changes in emotional states (Dawson et al., 2007). We suggest that discovering atypical patterns of ANS activity during speech and/or other behaviors in young children who stutter (CWS) may provide critical clues to the physiological processes occurring in early stuttering and their potential linkage to speech sensorimotor development. We hypothesized that SNS arousal affects speech motor planning and execution and plays a role in persistent stuttering in children (Smith and Weber, 2017). We also suggested that while SNS arousal may increase the likelihood of speech breakdowns, the occurrence of stuttering behaviors, in turn, may lead to increases in SNS activity (Weber and Smith, 1990; Smith and Weber, 2017). Therefore, an important first step to take experimentally is to determine if CWS show differences in sympathetic arousal associated with speaking or other tasks. Our goal in this experiment is to analyze physiological signals that reflect SNS function (e.g., electrodermal activity, blood pulse volume (BPV), and pulse rate) recorded during a range of experimental tasks performed by preschool CWS and children who do not stutter (CWNS). We also explore relationships among these physiological signals and selected behavioral assessments of stuttering and temperament.

The phrase “fight or flight” is synonymous with the SNS, while the contrasting term “rest and digest” is common shorthand for the functions of the other division of the ANS, the parasympathetic nervous system (PNS). Although this dichotomous description is an oversimplification of the complex and interrelated functions of the two branches of the ANS, it describes how the anatomically distinct sympathetic and parasympathetic branches of the ANS help maintain homeostasis by dynamically modulating internal physiological functions. The ANS is a mediator between brain and body. Efferent signals from the SNS, for example, trigger multiple simultaneous systemic responses—accelerated heart rate, blood vessel constriction, and increased perspiration in response to varied cognitive, motor, emotional, and other behavioral states (Andreassi, 2007). Afferent information regarding visceral states is relayed back to the central nervous system influencing our thoughts, emotions, and actions (Jänig, 2006; Cardinali, 2018). Autonomic contributions to motor, cognitive, and emotional behaviors occur through a neural matrix interconnecting the ANS with cortical, subcortical, and limbic areas with cingulate and insula cortex serving as putative interfaces (Oppenheimer et al., 1992; Fredrikson et al., 1998; Williams et al., 2001; Critchley, 2005, 2009; Pereira et al., 2010).

Electrodermal and blood pulse measures index SNS responses to diverse physiological and psychological triggers (Andreassi, 2007; Boucsein, 2013). The electrodermal signal is typically analyzed as two components. Tonic skin conductance level (SCL) is an index of sympathetic tone, reflecting slowly changing electrodermal activity (i.e., increased sweat secretion) over longer periods of time. SCL is useful for investigating general states of arousal or alertness (Dawson et al., 2007). In contrast, skin conductance responses (SCRs), smaller, transient changes, index changes in electrodermal activity occurring within 1–3 s of a specific event. SCRs are typically used as indices of attentional processes or stimulus significance (Dawson et al., 2007; Mendes, 2009). Higher amplitude SCLs/SCRs and more frequent SCRs indicate increased SNS arousal. Decreases in BPV amplitude through vasoconstriction also indicate SNS arousal, while increases in pulse rate primarily reflects SNS activity (Andreassi, 2007).

Increased sympathetic activity produces a wide range of physiological phenomena that could affect sensorimotor control indirectly, for example, changes in blood flow to muscles. Zimmermann (1980) proposed a direct mechanism by which sympathetic activity related to arousal could affect speech planning and execution in people who stutter. He noted preliminary evidence from animal studies demonstrating sympathetic efferent modulation of muscle spindles (mechanoreceptors embedded in muscles that provide proprioceptive feedback during movement) sensitivity. This suggests that SNS arousal could alter sensory signals generated during speech production. Subsequent studies in cat and rabbit confirmed the presence of sympathetic innervation of muscle spindles and demonstrated that sympathetic activation alters muscle spindle sensitivity to stretch and thus reflex excitability and central feedback signals (Roatta et al., 2002; Passatore and Roatta, 2006).

Radovanovic et al. (2015) provided anatomical evidence confirming the presence of sympathetic innervation of human muscle spindles. There are few physiological studies of alterations in proprioceptive sensitivity in response to sympathetic activation in humans (Passatore and Roatta, 2006). Results of one recent study are intriguing and suggest a potential linkage among emotional states, sympathetic activation, and sensorimotor processes. Ackerley et al. (2017) used microneurography to record activity of single muscle afferents in the human leg. They found that varying emotional states (elicited by music) resulted in increased sympathetic arousal (e.g., skin conductance) and changes in spindle sensitivity. We know that classically described muscle spindles are densely supplied in some muscles involved in speech (e.g., jaw closing muscles) but are absent in others (e.g., lip muscles) (Smith, 1992). In muscles that lack typical spindles, other mechanoreceptors provide proprioceptive information. In humans, although lip muscles lack typical spindles, lip proprioceptive sensitivity is equal to that of the jaw (Frayne et al., 2016). These findings support the notion that varying levels of sympathetic arousal could produce variability in sensory signals generated during speech and in the speech motor learning process, which ultimately, could affect the stability of speech motor programs.

There is also evidence that the SNS generates task-related efferent command signals during voluntary movement. In experiments in human subjects, Vissing and colleagues recorded microneurographic signals from skin sympathetic nerves during static hand grip tasks (Vissing et al., 1991). They showed that the skin sympathetic discharge preceded the onset of grip force increases and therefore is driven by central commands and not simply a response to muscle activation (Vissing et al., 1997). They reported that cutaneous sympathetic activation targeted eccrine (sweat) glands and vascular smooth muscle. A detailed review of this literature is not possible in this context, but higher levels of sympathetic arousal affect the control and coordination of simple grip movements as well as of complex movements, such as piano playing, skiing, and marksmanship (Noteboom et al., 2001; Vickers and Williams, 2007; Yoshie et al., 2009, 2016). In a study of increased sympathetic arousal on children’s gross motor control, stepping movement trajectories became less efficient and smooth under conditions in which children were observed compared with unobserved conditions (Beuter and Duda, 1985; Beuter et al., 1989). These findings suggest that during the initiation and performance of voluntary movement, there is temporal coupling of the outflow of central motor commands to muscles and SNS activation to skin.

There have been far fewer studies examining the relationship between sympathetic arousal and speech motor control. Kleinow and Smith (2006) assessed the spatiotemporal coordination of articulatory movements while typically fluent participants spoke aloud sentences under lower and higher arousal induced by a Stroop task. They found that the Stroop speaking condition was associated with increased SNS activity and higher speech coordination variability in school-age children and adults. In their study of the effects of arousal on SNS and voice indices, MacPherson et al. (2017) also used a Stroop speaking condition to increase arousal levels in healthy adults. They observed increased SCR amplitudes concomitant with changes in acoustic measures of voice under higher cognitive load. At a conceptual level, then, we suggest that in considering developing speech motor systems and stuttering, we should explore the potential role that speech-related central control of SNS discharge plays.

Two investigations of ANS activity in adults who stutter (AWS) and control participants (adults who do not stutter-AWNS) firmly support the conclusion that AWS and AWNS do not differ in average levels of sympathetic arousal during a range of experimental tasks. Peters and Hulstijn (1984) recorded subjective ratings of anxiety along with sensitive indices of ANS activity: heart rate, BPV, and electrodermal activity in 24 AWS and 24 AWNS before and during speech tasks (reading and conversation) and non-speech tasks (a motor and intelligence task). AWS reported higher anxiety levels than AWNS; however, there were no differences between the groups on any of the physiological variables. Heart rate, BPV, and electrodermal activity recorded before and during speech tasks were higher compared with the physiologic activity before and during non-speech tasks for both groups of speakers. Weber and Smith (1990) employed a similar experimental design with the aim of “scaling” ANS activity in speaking in relation to a set of tasks selected to elicit a range of sympathetic arousal from low (jaw wagging) to high (Valsalva maneuver). Electrodermal activity, BPV, and heart rate were recorded in 19 AWS and 19 AWNS and measured before, during, and after task performance. Similar to Peters and Hulstijn, we found that speaking was associated with relatively large increases in autonomic activity in both AWS and AWNS, and there were no differences between the two groups. From these two studies of adults, it seems reasonable to conclude that the act of speaking involves relatively high, but similar levels of sympathetic arousal in normally fluent adults and in AWS.

There have been a limited number of studies of the physiological correlates of ANS activity related to speech production in children. Studies from our laboratory suggested that normally fluent school-age children have higher levels of sympathetic arousal than adults during speech tasks (Arnold et al., 2014) and that increased sympathetic activation produced by a Stroop task results in higher variability in speech motor coordination for both children and adults (Kleinow and Smith, 2006). Within the framework of their Emotional Diathesis model of early stuttering, Walden, Conture and colleagues have completed a series of investigations in children using psychophysiological measures related to ANS functions (Walden et al., 2012). This group is the first to record ANS signals in preschoolers who are stuttering. Jones et al. (2014b) measured mean SCLs (indexing sympathetic activation) during baseline, listening, and speaking conditions, and found similar SCLs during baseline. They noted higher SCL in CWS while they viewed a positively valenced video clip, while CWNS had higher SCL while viewing a negatively valenced video clip. They also reported a group by condition effect in which CWS had higher SCL during a story-retelling task after viewing the positive video clip suggesting that CWS and CWS responded differently to tasks designed to induce different emotions.

Choi et al. (2016) recorded SCLs in 47 CWS aged 36–71 months during neutral baseline and while participants viewed positive and negative video clips. They used correlational analyses to investigate relationships among emotional reactivity, stuttering frequency, and sympathetic arousal. SCLs were not significantly correlated with the variables they examined, a finding they interpreted as not supporting the hypothesis that sympathetic arousal is a mediating variable between emotional stress and stuttering frequency. Zengin-Bolatkale et al. (2015) found higher SCLs in 3-year-old CWS compared to age-matched CWNS during a stressful picture naming task, although this finding did not hold for the 4- and 5-year-old age group comparisons. Both groups of children showed the expected increase in tonic SCL during task performance compared to baseline. As we have noted (Smith and Weber, 2017), the lack of stuttering/non-stuttering group differences does not necessarily imply that a particular factor is ultimately not significant for recovery versus persistence of stuttering. On this point, in a more recent study, Zengin-Bolatkale et al. (2018) reported that higher sympathetic arousal during the stressful picture naming task conducted when the children were in preschool was predictive of later, persistent stuttering.

Prior to these few investigations of the presumed physiological correlates of SNS arousal in CWS, earlier studies explored emotional development and temperamental factors in CWS through observational or parent-report measures. Temperament is defined as a biologically based construct encompassing emotional, attention, and motivational factors (Bates, 1989). A number of studies have used parent-report measures to investigate possible temperamental differences between CWNS and CWS. Several studies found that CWS are prone to increased anger or frustration, have greater difficulty regulating emotions, and are less able to adapt to change compared to CWNS (Anderson et al., 2003; Karrass et al., 2006; Eggers et al., 2010). However, other studies have not revealed temperamental differences between young CWS and CWNS using parent-report measures (Reilly et al., 2013; Kefalianos et al., 2014, 2017).

In summary, the potential role of sympathetic arousal in early stuttering and in its persistence is just beginning to be explored. Using a paradigm that we employed in earlier experiments to attempt to scale relative levels of autonomic arousal across tasks (Weber and Smith, 1990; Arnold et al., 2014), we examine task-related sympathetic arousal in preschool CWS and CWNS. Comparing sympathetic arousal in preschool children during speech and non-speech tasks, we hypothesized that CWS would show higher SNS activity compared to CWNS, particularly during speaking tasks. Given the recent interest in potential temperamental differences between CWS and CWNS (e.g., Anderson et al., 2003; Karrass et al., 2006; Eggers et al., 2010; Alm, 2014; Kefalianos et al., 2014, 2017), we included a parent-report measure of temperament (Putnam and Rothbart, 2006) and a self-report measure that assesses a child’s feelings about their communication (Vanryckeghem and Brutten, 2006). We examine relationships among physiological measures of SNS arousal and behavioral indices of temperament and communication attitude.

## Materials and Methods

### Participants

The research protocol was approved by the Institutional Review Board at Purdue University and adhered to Human Research Protection Program regulations and guidelines. We obtained written informed consent from parents or legal guardians, henceforth referred to as parents, at the beginning of the session. Seventy-two preschool children participated in the study, 47 CWS (38 boys and 9 girls, *M* = 55.9 months, *SD* = 7.6, range = 46–72) and 25 CWNS (18 boys and 7 girls, *M* = 55.6 months, *SD* = 6.3, range = 49–70). Parents reported that their child did not have a developmental, cardiovascular, or neurological disorder and confirmed that North American English was their child’s first and primary language. Parents also noted whether their child had normal or corrected-to-normal vision, had not consumed caffeine prior to the experiment, and were not taking medications affecting the central nervous and cardiovascular systems (e.g., depressants, stimulants, analgesics, anticoagulants etc.). All participants scored within normal limits on assessments of non-verbal intelligence (PTONI; Ehrler and McGhee, 2008) and social development (Childhood Autism Rating Scale, 2nd Edition CARS-2; Schopler et al., 2010) and passed a bilateral pure-tone hearing screening at 500, 1000, 2000, 4000, and 6000 Hz at 20 db. Finally, the two groups had comparable socioeconomic status (SES) (CWS *M* = 5.60, *SD* = 1.02; CWNS *M* = 6.20, *SD* = 0.65) determined by the parent’s highest level of education (Hollingshead, 1975). SES was evaluated on a 7-point scale (1 = *less than 7th grade education* to 7 = *completion of a graduate or professional degree*).

### Stuttering Diagnosis

We diagnosed childhood stuttering using standardized and observational measures. First, we collected spontaneous speech samples from each child during two play sessions, one with their primary caregiver, and the other with the project speech-language pathologist (SLP), for a combined total of 750–1000 syllables. We calculated a weighted stuttering index (WSI) for each child based upon the frequency of part- and single-syllable word repetitions, the number of iterations, and the presence and duration of dysrhythmic phonations per 100 syllables of spontaneous speech (Ambrose and Yairi, 1999). A score of 4.0 or higher indicates stuttering. We also administered the Test of Childhood Stuttering (TOCS; Gillam et al., 2009), a norm-referenced assessment tool to each child. An index score of 84 or below on this assessment identifies the child as stuttering.

In addition, the parent and SLP with experience in fluency disorders rated the child as stuttering by assigning them a score of 2 or higher on an 8-point scale [0–1 = normal; 2–3 = mild stuttering; 4–5 moderate stuttering; 6–7 = severe stuttering]. The clinician’s rating was based upon the type, duration, and frequency of disfluencies along with the presence and severity of secondary characteristics while considering the child’s own awareness and/or anxiety about his or her disfluencies.

Three CWS had a WSI <4.0; however, we retained these children in the study because they met the criteria for stuttering based upon the TOCS and clinician and parent ratings. The average duration of stuttering (i.e., time since onset) was 21 months (*SD* = 11 months) according to parent report.

### Experimental Stimuli and Procedures

The experiment comprised speech and non-speech tasks that were adapted for research with young children from our earlier studies with older children and adults (Weber and Smith, 1990; Arnold et al., 2014). There were four tasks: open/close jaw wag (lower effort, non-speech condition), structured sentence production (lower effort, speech condition), picture description (higher effort, speech condition), and forceful blowing (maximal maneuver, non-speech condition). The order of the jaw wag, sentence production, and picture description tasks was counterbalanced across subjects; however, we consistently collected a baseline at the beginning (pre-baseline) and a baseline at end of the experiment (post-baseline) before the blowing task. Forceful blowing, designed to elicit a maximal response, was always collected at the end of the experiment so that the expected high levels of autonomic arousal would not influence data collected during the other experimental tasks. Before each task, we explained and demonstrated what the child would do, then had each child practice two to three trials to ensure they understood the task. During data collection, a 15 s rest preceded each of the four tasks, and a short break was taken at the end of each task. During these breaks, the participants either collected a sticker or took a turn at a game.

For the two resting baseline intervals collected at the beginning and near the end of the experiment, the children saw a picture of a child resting outdoors with her eyes closed and were encouraged, but not required, to close their eyes, be still, and rest for 1 min. For the first 13 CWS who participated in the experiment, these initial and final baselines were collected in a different manner. In these cases, baseline intervals were interspersed within the experiment before each task. However, we found that these inter-task intervals were not sufficiently long enough to allow sympathetic arousal to return to resting levels and were often contaminated by movement artifacts. Our statistical approach, described below, accounts for these missing baseline measures from the 13 CWS.

The experimental tasks consisted of 2 speech and 2 non-speech tasks:

Jaw wag (JAW). For this non-speech task, the children continuously opened and closed their mouth for 6 s while watching an animated face opening and closing its mouth, then rested for 5 s. We collected two trials of five jaw wag intervals each.

Structured sentence production (SENT). For this speech task, the children saw pictures of familiar objects or animals inside a box or a barn and spoke aloud the simple, declarative carrier sentences, “The____is in the barn” or “The____is in the box.” We considered this task less demanding in terms of required language formulation (Arnold et al., 2014). The children produced three sentences in a row to identify what they saw in three different slides (e.g., “The cookie is in the box…The doll is in the box…The ball is in the box”) then rested for 5 s before the next sequence of three picture slides appeared. Each child completed at least two trials with five sets of three sentences in each.

Picture Description (PIC). During this speech task, the children viewed child-friendly black and white picture scenes and described what was happening in each one. One experimenter modeled the task for two scenes then had each child practice describing two scenes so that they understood the task and could describe the picture scenes using connected speech (e.g., “There’s a farmer on a tractor”, as opposed to listing items (e.g., farmer…tractor…cow) that they recognized. The experimenters controlled the rate of picture presentation to ensure that we collected several utterances per scene. If children did not respond, or produced only a brief response, the experimenter encouraged them by saying, “Tell me more about that” or “What else is going on in the picture?” Within a trial, each picture scene was separated by 5 s of rest. The children completed at least two trials of five picture scenes for a minimum of ten picture scenes. If children produced few responses or were disfluent, we presented an additional block of three picture scenes.

Forceful blowing (MAX). Participants completed a forceful blowing task included to elicit relatively high levels of sympathetic arousal. In many experiments, a Valsalva maneuver is used establish a maximal response (Boucsein, 2013). Through pilot testing, we found that young children could not reliably produce a Valsalva, so they blew on a party blower and held it extended against the experimenter’s hand for 3–5 s. Each child completed two trials of three to five blows.

### Data Acquisition

SNS signals were collected with a Biopac MP150 data acquisition system running AcqKnowledge 4.4 acquisition software. The system included a Biopac GSR100C amplifier to measure electrodermal activity and a PPG100C pulse plethysmograph amplifier for BPV measures. After completing an adapted handedness inventory (Oldfield, 1971), the children were seated at a small desk while the experimenter affixed pre-gelled self-adhesive Ag/AgCl electrodes to the hypothenar eminence and thenar eminence of their non-dominant hand to record electrodermal activity. We recorded skin conductance in microSiemens (μS) between the two electrodes at a 2.5 kHz sampling rate with an initial gain of 10 μS/V and low-pass filtered at 1 Hz. Next, a photoplethysmograph transducer (Biopac TSD200) was secured around the distal phalanx of the fourth finger of the child’s non-dominant hand with a Velcro strap. An infrared emitter and photodiode detector embedded in the transducer measured the relative changes in blood flow. The BPV signal was collected with a gain of 50 at a 2.5 kHz sampling rate.

We presented the experimental stimuli (e.g., pictures, animations) in Microsoft PowerPoint on a 25 in monitor. The participants’ speech acoustic signal was collected with a Shure SM90 tabletop condenser microphone at 10 kHz, and video recordings of the experimental session were made with a Logitech HD 720p webcam. The audio and video signals were synchronized with the autonomic recordings through the Biopac MP150 acquisition system. These signals were used for off-line transcription and to ensure that segments used in subsequent analyses were free from movement artifact, unrelated comments, or redirection from the experimenter.

### Signal Processing and Analysis

**FIGURE 1:**
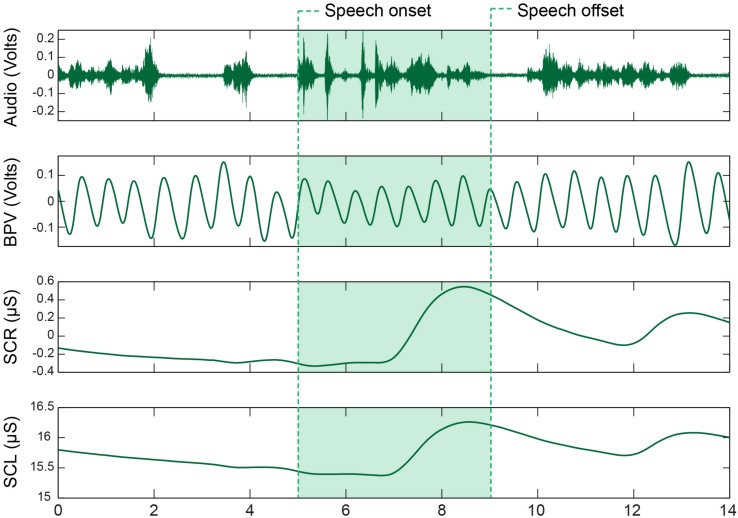
Sympathetic nervous system (SNS) recordings from a CWS during the picture description task. The waveforms represent, from top to bottom, the acoustic signal, blood pulse volume (BPV) signal, phasic skin conductance response (SCR), and tonic skin conductance level (SCL). Utterances were extracted from the long recording by identifying speech onsets and offsets within the acoustic record.

After the experiment, the physiological signals were exported into MATLAB (ver. 2015a). The electrodermal signal was downsampled to 250 Hz to reduce file size. We derived SCR measures from SCL via high-pass filtering (Fpass 0.07 Hz/Apass 0.5 dB). The BPV signal was downsampled to 250 Hz and digitally band pass filtered between 0.5–3 Hz with a 100-order FIR filter (Arnold et al., 2014). We downsampled signals to reduce file sizes and subsequent processing time and because the energy in these SNS signals is low frequency. A custom MATLAB program simultaneously displayed the acoustic record, BPV signal, and SCL and SCR signals on the monitor from a trial (Figure 1). The synchronized video record was displayed on an adjacent monitor. As shown in Figure 1, the experimenter extracted SCL, SCRs, and BPV segments from the longer, continuous recordings by indicating onset and offset points in the acoustic record with a cursor. Other studies have recorded SCL over longer, several minute epochs (e.g., Jones et al., 2014b). This presents challenges for studies with preschool children as movement artifact, redirection from the experimenter, or unrelated comments from the child may inflate indices of sympathetic arousal. For pre and post baselines, we extracted 5 s intervals from the 1 min records when the child was still, quiet, and electrodermal activity reached a minimum. For the JAW task, we selected 4–6, 6 s trials of accurate jaw wagging from each participant. We analyzed exclusively fluent (non-stuttered) productions from the two speaking tasks, SENT and PIC, in order to make a valid comparison of sympathetic activity between CWS and CWNS. For the SENT task, we selected 4–9 fluent speech segments. These segments included all three sentences produced in a row or in some cases, 1–2 sentences if a sentence was unusable due to movement artifact, for example. Given that SNS responses typically occur within 1–3 s of a stimulus (Lim et al., 2003; Andreassi, 2007; Dawson et al., 2007), we selected fluent utterances that were not immediately adjacent (within ∼3 s) of a stuttering-like disfluency for the speech tasks. We segmented connected speech into utterances or verbal productions bounded by grammatical closure, intonation contours, or long pauses following Systematic Analysis of Language Transcripts (SALT) conventions (Miller and Iglesias, 2006). Onset/offset indices for the tasks were adjusted so that segments selected for analysis did not contain motion and other artifact. Finally, for the MAX task, we selected the first three successful trials in which the child inhaled and blew out on the party blower holding it extended for at least 3 s.

#### Reliability

For baselines and JAW and MAX tasks, intervals and trials were marked usable during data acquisition and confirmed later during offline data analysis. The first author reanalyzed 20% of the data from the SENT (20 trials from CWS and 9 trials from CWNS selected at random) and PIC (25 trials from CWS and 12 trials from CWNS selected at random) tasks to establish inter-rater reliability for fluent utterance coding (whether utterances selected for analysis did not contain instances of stuttering, aside comments, or movement). The percentage of coding agreement was 99% for SENT and 97% for PIC indicating excellent reliability.

### Dependent Measures

#### Electrodermal Measures

We analyzed 3 electrodermal measures: (1) SCR amp, the amplitude of the phasic response, calculated by subtracting the minimum SCR value from the maximum SCR value associated with the first SCR peak (if any) within the window of analysis. Increases in SCR amp indicate phasic increases in conductivity between the electrodes due to increased sweat secretion associated with increased SNS arousal (Boucsein, 2013). (2) SCR frequency, the number of SCR responses (≥0.05 μS threshold) within the window of analysis. An increased number of SCRs is also associated with increased SNS arousal. (3) SCL, the average tonic or slower changing index of electrodermal activity. Higher SCL is indicative of increased SNS arousal. We computed the average SCR amp, SCR frequency, and SCL from trials associated with a particular task.

#### Blood Pulse Volume Measures

We analyzed two BPV measures: (1) BPV amp, the average trough-to-peak amplitude (in volts) of pulse cycles across each segment from a particular task was calculated using an automatic peak-detection algorithm following procedures from Arnold et al. (2014). Decreases in BPV amplitude via vasoconstriction signifies SNS arousal. (2) Pulse rate (PR in pulses per minute-ppm), a direct measure of heart rate, was recorded for each segment then averaged for each task. Increases in PR generally indicate increased SNS arousal.

#### Task Performance Measures

It is important to note that electrodermal and blood pulse responses are not elicited during all trials (Andreassi, 2007; Boucsein, 2013). We selected trials for analysis based on when the child was on task and sitting still regardless of whether there was a clear SNS response. We compared behavioral performance on the two speaking tasks by calculating average syllable counts for responses on the PIC task and average speaking rate (syllables/s) for both the PIC and SENT tasks. Typical disfluencies, for example, filled pauses (um/uh) or multisyllabic word and phrase repetitions were tabulated for the PIC task, but not included in the syllable count. We included these analyses to assess whether potential group differences in SNS recordings were driven, for example, by differences in speaking rate and/or language formulation.

#### Children’s Behavior Questionnaire and KiddyCAT

We obtained a temperament profile for each child using the short form of the Children’s Behavior Questionnaire (Putnam and Rothbart, 2006). The CBQ is an established measure of temperament based on parental report and has been used to assess temperament characteristics in CWS in previous studies (see review in Jones et al., 2014a). The parent identified as the child’s primary caregiver completed the questionnaire. The short form CBQ contains 94 items that assess 15 temperament dimensions. The parent responded to each item using a 7-point Likert scale with scores ranging from 1 or “extremely untrue of your child” to 7 “extremely true of your child.” If the parent had never observed their child in a particular situation they could answer “not applicable” to that item. A child’s scores across the 15 temperament scales are then combined into three composite personality scores: (1) positive emotional reactivity or surgency/extraversion (2) negative emotional reactivity or negative affectivity, and (3) effortful control or self-regulation of reactivity and attention of reactivity and attention. Finally, we also administered the KiddyCAT to each participant (Vanryckeghem et al., 2005; Vanryckeghem and Brutten, 2006). This measure assesses a preschool child’s attitude about their communication through 12 yes/no questions. The maximum score on the KiddyCAT is 12; children who receive higher scores on this measure are considered to have more negative feelings about their speaking abilities.

### Statistics

#### Physiological Data

Statistical analyses were conducted in Mplus version 7.4. To account for the missing pre-experiment resting baselines from 13 of the CWS, regression models were estimated using direct maximum likelihood estimation to include observations with missing items (Arbuckle, 1996; Enders and Bandalos, 2001). The nesting of repeated measures within children was accounted for using clustered standard errors (Binder, 1983). We examined sex as a factor in preliminary models; however, sex effects in these models were not significant, so data from boys and girls was pooled in each participant group. The statistical models controlled for both child age (in months) and baseline levels of each of the five SNS variables: SCR amp, SCR frequency, SCL, BPV amp, and PR. Marginal model means were obtained for specific group and task combinations. A single test of any difference in the five SNS variable means across the four tasks was calculated and specific differences in variable means across every combination of tasks were assessed. A Bonferroni correction was applied to the six specific pairwise task means tests (*p* ≤ 0.008). We used partially standardized beta coefficient estimates for effects sizes which are comparable to Cohen’s *d* estimates. Similar to Cohen’s *d*, these effects sizes provide the estimated mean difference in outcomes in standard deviation units; however, they are conditioned on the covariates in the model. Standard interpretation of this index: ES of 0.20 = small effect, ES of 0.50 = moderate effect and ES of 0.80 = large effect was applied.

#### Behavioral Data

We used modified *t*-tests that controlled for child age to compare the two groups’ performance on the following measures: speaking rate and syllable count for the SENT and PIC tasks, the KiddyCAT, and the three CBQ composite scores: extraversion, negative affectivity, and effortful control. We computed regression analyses on the dataset from CWS to examine the relationship between stuttering severity (WSI scores) or CBQ scores and the five physiological SNS variables from the PIC task (i.e., SCR amp, SCR frequency, SCL, BPV amp, and PR). In these analyses, we computed regressions using WSI scores and CBQ composite scores as predictors and SNS variables for the PIC task as outcomes.

## Results

### SNS Measures

#### Group Effects

**FIGURE 2:**
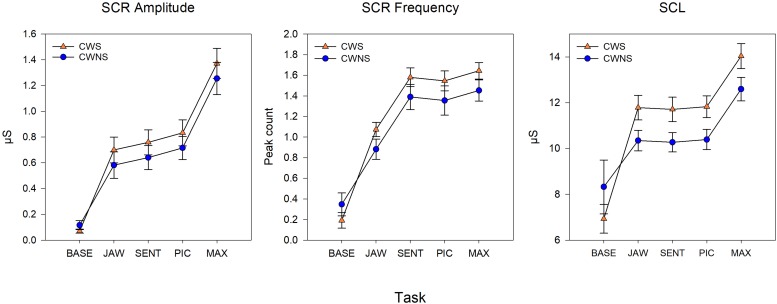
Model means and standard error estimate bars for skin conductance measures from each group plotted by task. The graphs show the skin conductance response amplitude (left), skin conductance response frequency (middle), and tonic skin conductance level (SCL) (right). BASE, initial baseline; JAW, jaw opening/closing; SENT, structured sentence production; PIC, picture description task; MAX, blowing/maximal maneuver.

Statistical results including beta coefficients resulting from regression analysis that indicate mean differences in dependent variables for a unit change in predictor variables (e.g., group mean differences), *p*-values, effect sizes (beta coefficients with standardized outcomes (i.e., group differences for outcomes in standard deviations units), and confidence intervals for the five SNS variables are listed in Table 1. The means with standard error bars for skin conductance and blood pulse measures for each group are plotted in Figures 2, 4, respectively.

**TABLE 1:** Statistical results from regression with group, task, and covariate coefficients.

	**SCR Amp**				**SCR Frequency**				**SCL**				**BPV Amp**				**PR**			
					
	***b***	***p*-value**	**ES**	**CI**	***b***	***p*-value**	**ES**	**CI**	***b***	***p*-value**	**ES**	**CI**	***b***	***p*-value**	**ES**	**CI**	***b***	***p*-value**	**ES**	**CI**
**GROUP**																				
CWS vs. CWNS	0.12	.38	0.16	(−0.14, 0.38)	0.19	.13	0.27	(−0.05, 0.44)	1.44	**.03^∗^**	0.24	(0.18, 2.70)	–0.25	.19	–0.25	(−0.62, 0.12)	0.21	.92	0.02	(−3.66, 4.09)
**TASK**																				
Jaw vs. Max	–0.67	≤.008^∗^	–0.90	(−0.87, −0.48)	–0.57	≤.008^∗^	–0.81	(−0.71, −0.43)	–2.25	≤.008^∗^	–0.37	(−2.83, −1.66)	0.90	≤.008^∗^	0.91	(0.73, 1.08)	4.10	≤.008^∗^	0.38	(2.12, 6.09)
Sent vs. Max	–0.61	≤.008^∗^	–0.83	(−0.80, −0.43)	–0.06	.49	–0.09	(−0.25, 0.12)	–2.32	≤.008^∗^	–0.38	(−2.97, −1.68)	0.97	≤.008^∗^	0.98	(0.78, 1.16)	7.67	≤.008^∗^	0.71	(6.06, 9.28)
Pic vs. Max	–0.54	≤.008^∗^	–0.73	(−0.72, −0.36)	–0.10	.36	–0.14	(−0.31, 0.11)	–2.21	≤.008^∗^	–0.36	(−2.82, −1.59)	1.03	≤.008^∗^	1.04	(0.85, 1.20)	7.79	≤.008^∗^	0.72	(5.94, 9.64)
Jaw vs. Sent	–0.06	.31	–0.08	(−0.17, 0.06)	–0.51	≤.008^∗^	–0.72	(−0.65, −0.36)	0.08	.78	0.01	(−0.45, 0.60)	–0.06	.35	–0.07	(−0.20, 0.07)	–3.57	≤.008^∗^	–0.33	(−4.73, −2.41)
Jaw vs. Pic	–0.13	.03	–0.18	(−0.26, −0.01)	–0.47	≤.008^∗^	–0.67	(−0.63, −0.32)	–0.04	.88	–0.007	(−0.61, 0.53)	–0.12	.03	–0.12	(−0.24, −0.01)	–3.68	≤.008^∗^	0.65	(−4.96, −2.40)
Sent vs. Pic	–0.08	.15	–0.10	(−0.18, 0.03)	0.04	.69	0.05	(−0.13, 0.20)	–0.12	.65	–0.02	(−0.63, 0.39)	–0.06	.38	–0.06	(−0.19, 0.07)	–0.12	.82	–0.01	(−1.10, 0.87)
**COVARIATE**																				
Baseline	1.14	.06		(−0.07, 2.35)	0.48	≤.01^∗^		(0.22, 0.74)	1.07	≤.01^∗^		(0.92, 1.23)	0.25	≤.01^∗^		(0.08, 0.41)	0.24	≤.01^∗^		(0.07, 0.42)
Age (mos.)	0.009	.32		(−0.03, 0.008)	0.01	.09		(0.00, 0.03)	–0.08	.03^∗^		(−0.16, −0.01)	–0.01	.56		(−0.03, 0.01)	–0.39	≤.01^∗^		(−0.65, −0.13)

**b*, beta value; Effect size (ES) are the standardized beta coefficients; CI, confidence interval. Asterisks denote statistically significant results.*

**FIGURE 3:**
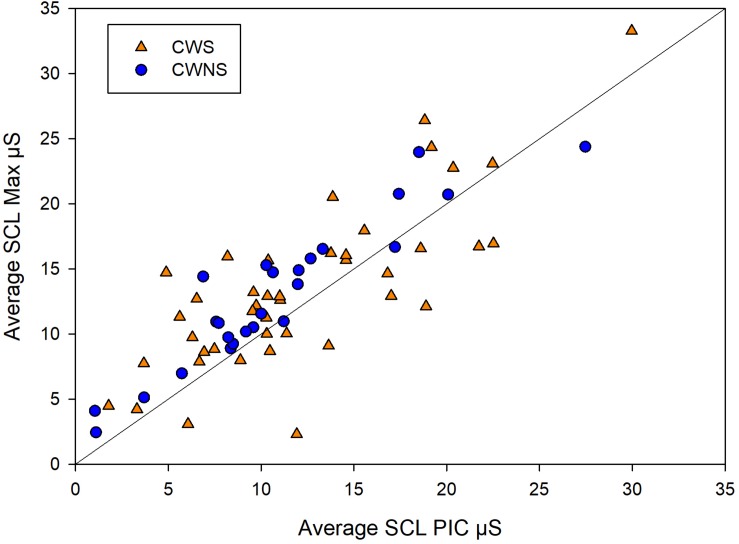
Individual points from CWS (triangles) and CWNS (circles) of a participant’s average PIC skin conductance level (SCL) plotted against their average MAX SCL and identity line. Units are in microSiemens. These raw means are slightly different from the estimated marginal means used in the statistical models reported in the Results section.

Figure 2 shows the average SCR amp, SCR frequency, and SCL for each group by task. On average, CWS had slightly higher SCR amp (left graph) and SCR frequency (middle graph) compared to CWNS across tasks, but this difference was not statistically significant (Table 1). However, the CWS had significantly higher mean SCLs across tasks compared to CWNS (but not at baseline) as shown in the right graph of Figure 2. Participant’s SCLs were highly correlated across experimental tasks. Figure 3 shows participants’ average SCL for PIC plotted against their average SCL for MAX. SCL for the two tasks showed a strong correlation (*r* (72) = .83, *p* < .001 when both groups were combined. Although the range in values for participants in each group overlap, compared to CWNS, more CWS fall below the identity line (y = x), or have higher PIC SCLs than MAX SCLs. We calculated that 11/47 or 23% of CWS and 1/25 or 4% of CWNS had PIC SCL > MAX SCL (difference ≥ 1 μS). These are the CWS participants whose data points are below the identity line in Figure 3.

**FIGURE 4:**
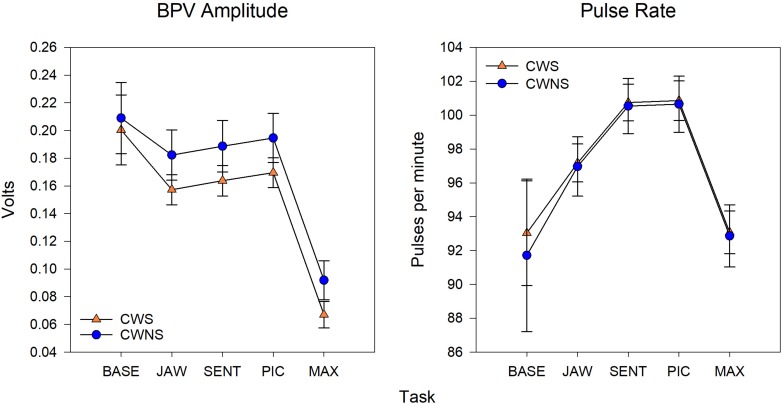
Model means and standard error estimate bars for blood pulse volume (BPV) amplitude (left graph) and pulse rate (right graph) from each group plotted by task. BASE, initial baseline; JAW, jaw opening/closing; SENT, structured sentence production; PIC, picture description task; MAX, blowing/maximal maneuver.

Figure 4 shows the average BPV amp and PR by task for each participant group. On average, CWS had smaller BPV amplitudes than CWNS across tasks (see left graph of Figure 4); however, the difference in means across tasks was not statistically significant (Table 1). Finally, average PRs for each group were nearly identical across tasks (right graph of Figure 4).

#### Task Effects

The Group X Task interactions were not significant for SCR amp, SCR frequency, SCL, BPV amp, or PR in our initial statistical models. Therefore, data from CWS and CWNS were pooled to examine task effects. A statistical summary of these effects is provided in Table 1.

As expected, SCR amp for MAX was significantly higher than the other tasks (Figure 2). Although the comparison between JAW and PIC approached significance, it did not survive Bonferroni correction, and no other comparison was significant. SCR frequency was significantly higher for MAX, SENT, and PIC tasks compared to JAW. Finally, significantly higher SCLs were reached for MAX compared to the other tasks (right graph of Figure 2).

As shown in the left graph of Figure 4, significantly smaller BPV amps were elicited during MAX compared to the other three tasks. The right graph in Figure 4 shows PR by task. The lowest PRs were elicited during MAX compared to the other three tasks, and PRs during JAW were significantly lower than PRs obtained during both the SENT and PIC tasks.

#### Covariate Effects

Baseline values of a participant’s SCR frequency, SCL, BPV amp, and PR, predicted their SCR frequencies, SCL, BPV amps, and PRs elicited by the tasks (Table 1). The effect of age in the model was significant for both SCL and PR. For SCL, a 1-month increase in age was associated with a decrease in SCL of approximately 0.08 μS. For PR, a 1-month increase in age was associated with a decrease in PR of approximately 0.39 ppm.

### Behavioral Measures

#### Group Performance Measures for SENT and PIC Tasks

We compared the CWS and CWNS performance on the two speaking tasks: SENT and PIC using modified *t*-tests that controlled for age. The two groups had statistically similar syllable counts for the PIC task (CWS: *M(SD)* = 11.70(2.65); CWNS *M(SD)* = 11.92(2.92) (standard. diff. = −0.11, *p* = 0.80). We also did not detect significant group differences in speaking rate, measured in syllables/s for either the SENT task (CWS: *M(SD)* = 1.50(0.26); CWNS *M(SD)* = 1.57(0.19) (stand. diff. −0.13, *p* = 0.20) or the PIC task (CWS: *M(SD)* = 1.57(0.29); CWNS *M(SD)* = 1.58(0.34) (stand. diff. −0.03, *p* = 0.81). Finally, we did not detect a group difference in the number of typical disfluencies that occurred during the fluent utterances extracted for the PIC task (CWS: *M(SD)* = 3.38(3.38); CWNS *M(SD)* = 3.32(2.70) (standard. diff. = −0.45, *p* = 0.19).

#### Group Results for CBQ and KiddyCAT

Means and standard deviations for the three CBQ composite scores and the KiddyCAT are listed in Table 2. The last row of this table provides the *p*-values and confidence intervals of the modified *t*-tests assessing between group differences on each measure. Overall, we obtained similar CBQ composite scores for CWS and CWNS; between group comparisons for the three CBQ composite scores: extraversion, negative affectivity, or effortful control were not significant. The CWS scored significantly higher than CWNS on the KiddyCAT indicating that they may harbor more negative feelings toward communication, although the overall means we obtained for both groups of children (Table 2) fell below the means reported for this measure (Vanryckeghem and Brutten, 2006; CWS *M* = 4.36; CWNS *M* = 1.79).

**TABLE 2:** Means and standard deviations for behavioral data with between-group comparison results from modified^∗^ *t*-tests.

	**CBQ**			**KiddyCAT**
		
	**Extraversion**	**Negative affectivity**	**Effortful control**	
CWS Mean (*SD*) *n* = 47	4.76 (0.72)	4.10 (0.88)	5.36 (0.48)	2.21 (2.40)
CWNS Mean (*SD*) *n* = 25	4.80 (0.61)	4.10 (0.77)	5.38 (0.53)	0.92 (1.47)
**Between-group**				
Standardized differences *p*-value; confidence interval	−0.03 *p* = .80; [−0.25, 0.19]	−0.03 *p* = .78; [−0.27, 0.20]	−0.03 *p* = .82; [−0.27, 0.21]	0.26 (*p* < .01)^∗∗^ [0.08, 0.43]

*^∗^Controlling for age; ^∗∗^significant at *p* < 0.01 level.*

#### Relationships Among Physiological and Behavioral Variables

**FIGURE 5:**
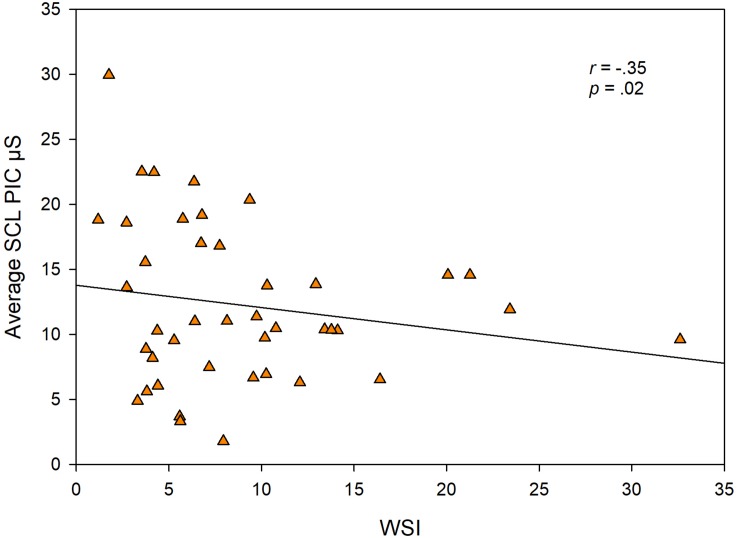
Individual CWS data points showing each child’s WSI score plotted against their average skin conductance level (SCL) from the PIC task.

To test the hypothesis that higher SNS arousal levels are associated with stuttering severity, we examined the relationship among SCL, SCR amp from the more demanding speech task (PIC) and stuttering severity (WSI score). TOCS and KiddyCat scores did not show a sufficient range to explore meaningful correlations, thus they were not included in the analysis. We did not find a significant relationship between WSI score and SCR amp (β = −0.24; *p* < .12), but found an unexpected significant negative relationship between the WSI score and PIC SCL (β = −0.35; *p* = .02). Figure 5 shows each CWS’s WSI score plotted against their average SCL for the PIC task.

Finally, we assessed the relationships for these same skin conductance measures from the PIC task with CBQ composite scores, extraversion, negative affect, and effortful control. None of these regression analyses revealed any significant correlation between the skin conductance measures and the three dimensions of temperament.

## Discussion

Overall, the results of our study do not support the hypothesis that atypically high levels of sympathetic arousal are associated with speech production in preschool children who are stuttering. There were no significant differences between groups in measures of transient skin conductance responses, BPV amplitudes, or in pulse rate. We did observe significantly higher background, tonic SCLs in CWS, on average, but these were not exclusive to speech production tasks. It is important to reiterate that the present results were derived from fluent intervals of speech, and SNS correlates of stuttering behaviors are reported in a companion paper (Walsh and Usler, in press).

To obtain an estimate of the participants’ resting, baseline levels of sympathetic activation, physiological data were recorded while the children sat quietly before performance of any of the experimental tasks (the issue of baseline is an important one, which we will discuss in more detail below). Based on the results we obtained showing clearly lower levels of autonomic activation in the resting baseline condition, this approach was successful. As expected, resting levels for all variables were lower compared to those observed during performance of all experimental tasks (see Figures 2, 4). Importantly, there were no differences between CWS and CWNS in baseline indices of SCL, SCR amp, SCR frequency, BPV or PR. This overall pattern of results across the two groups of participants and the systematic task effects we observed give us confidence in our conclusions regarding preschool children’s sympathetic activity levels during performance of this set of tasks.

### Central Circuits and SNS Control

Skin conductance changes reflect the activation of sympathetic cholinergic neurons innervating the eccrine dermal sweat glands, and they are sensitive indices of the modulation of arousal during emotional, cognitive, and physical behaviors (Venables and Christie, 1980). SCLs vary in the time span of tens of seconds, while SCRs, transient changes in skin conductance seen as rapid increases superimposed on the tonic background level, vary approximately 1–3 s (Dawson et al., 2007). Investigators have recorded SCL and SCRs during fMRI scanning while young adults used biofeedback to elevate or lower SCL (Nagai et al., 2004). Findings from this and other studies suggest that cortical systems with inputs to brainstem circuits are active in controlling background SCL and generating transient SCRs (Nagai et al., 2004; Critchley, 2005). Our results demonstrate that CWS have higher SCL during task performance compared to their fluent peers; whether this higher background SNS activity reflects higher levels of attention and focus on the tasks (Iani et al., 2004), higher performance anxiety (e.g., Simpson et al., 2001) or some other factor, we do not know. We also found a high degree of heterogeneity among the CWS in their levels of SCL during task performance, with most participants’ data overlapping that of the CWNS (Figure 3).

In an experiment designed to manipulate emotional responses to positive and negative video clips in preschool CWS and CWNS, Jones et al. (2014b) also measured SCL. The pattern of results was mixed when CWS were compared with CWNS across positive and negative viewing tasks. However, in the cases in which significant between group differences were observed, SCL was higher for CWS. We did not find significant differences in SCR amplitude or SCR frequency between the two groups, although CWS showed a trend for slightly higher SCR amplitude and frequency. There is evidence that the generation of SCRs is regulated through circuits involving cingulate, insula, right parietal lobe, motor cortex, and ventral medial prefrontal cortex (Nagai et al., 2004). Of course these imaging studies involved adult participants, and whether or not four and five-year-old children engage similar neural circuits in autonomic regulation is unknown. We cite this literature here simply to emphasize that the easily recorded signal indexing increased conductivity through eccrine sweat gland activity is, in fact, driven by complex cortical and brainstem networks.

We turn to the hypothesis advanced in the introduction that SNS activation co-occurs with normal control of movement and that excess sympathetic activation could be a contributor to motor instability in early stuttering. Most of the autonomic indices we assessed did not differ between the two groups across tasks. The fact that we observed higher levels of SCL across all tasks and that the overall pattern of relative SCL amplitude was similar for the groups of CWS and CWNS does not lend support to the idea that the neural activity driving higher SCLs is related to speech production. One hypothesis to consider is that speech specific increases in SNS activity develops later in CWS and/or that speech-specific phasic increases in SNS arousal would more likely be observed surrounding stuttering events. We addressed the latter hypothesis in a companion study (Walsh and Usler, in press). We found that stuttered utterances produced during a picture description task by CWS were associated with significantly higher phasic SCR amplitude, SCR frequency, and reduced BPV compared to fluent utterances. This suggests that transient sympathetic responses increase in intervals surrounding stuttering behaviors and therefore reflect changes in fluency states. Future studies will need to consider the potential bidirectional influences between SNS and speech neural control systems that can change dynamically during speech production driven by a variety of factors both internal and external to the speaker.

### Developmental Profiles of SNS Activity

Previous studies from our group and others provide some clues on a lifespan perspective on SNS activity related to speech production in stuttering and normally fluent speakers. Early investigations (Peters and Hulstijn, 1984; Weber and Smith, 1990) demonstrated that for AWS and AWNS, speech production tasks generally produced the highest levels of SNS arousal compared to difficult mental arithmetic (Peters and Hulstijn, 1984) and an effortful physical task (Valsalva maneuver, Weber and Smith, 1990). These results from adult participants contrast with those of most of the preschool children. Both CWS and CWNS typically produced the highest levels of sympathetic arousal during the forceful blowing task, which we designed to mimic the Valsalva maneuver. With the exception of SCR frequency (which was significantly higher for the two speech tasks, SENT and PIC, and MAX), maximal values of SCL and SCR amplitude and minimum values of BPV amp (all indicators of increased SNS activation) typically occurred in the MAX blowing task. In fact, for both groups of children, SCR amplitude almost doubled in the MAX condition compared to the two speech conditions (SENT and PIC, Figure 2). Weber and Smith (1990) show similar plots for groups of AWS and AWNS (their Figure 3), indicating SCR peaks were much smaller in amplitude compared to the preschoolers in this study and were approximately equal across the speech and Valsalva conditions. We actually observed lower SCL in both groups of adults in the Valsalva compared to the speech conditions. Taken together, these results suggest that the relatively high levels of sympathetic activation occurring in adults prior to and during speech, indicative of tight coupling between sympathetic arousal and speaking, emerges with maturation. Most preschool participants showed the highest sympathetic activation to a physically demanding task rather than to the more cognitively demanding picture description task. For a subgroup of the CWS, however, this typical pattern of highest SNS activation in the MAX task was not observed. Approximately one-quarter of CWS showed higher SCL levels in the picture naming task compared to the MAX task (Figure 3). This suggests that some CWS are atypically early in developing the adult pattern of higher levels of arousal during speaking compared to physically demanding tasks. This preliminary result is worth further exploration, as it will be important to determine whether a relationship exists between early developing speech motor/sympathetic outflow and stuttering persistence and recovery.

Also relevant to the issue of a lifespan perspective on autonomic regulation related to speech, we found no differences in pulse rate between the two groups of children. The highest PRs were observed in the two speaking conditions, while lower PRs were achieved for the non-speech conditions (Figure 4). PR reflects both sympathetic and parasympathetic control of heart rate, suggesting that CWS and CWNS did not differ in sympathetic/parasympathetic control of PR during fluent speech production. Jones et al. (2014b) used respiratory sinus arrhythmia (RSA) derived from heart rate variability as an exclusive index of parasympathetic “tone” in a study of preschool CWS and CWNS (cf. Grossman and Taylor, 2007). They found reduced baseline differences in RSA in CWS, evidence they interpreted as placing CWS at greater vulnerability to emotional reactivity. Our PR results also contrast with findings from adults. Alm (2004) reviewed the available literature on heart rate and speech production tasks in AWS and concluded that a decrease in heart rate is associated with speech tasks in AWS. Increases in heart rate are primarily associated with increased SNS arousal. Alm interpreted this finding as support for the hypothesis that speech-related anxiety produces a “freezing response” associated with parasympathetic reduction in heart rate. We note that both of these prior studies included stuttered and fluent speech in their analysis. In order to compare SNS arousal during speech production in CWS and CWNS, we focused exclusively on fluent utterances. We examined PR during fluent and stuttered speech production in a separate study (Walsh and Usler, in press). Overall, these findings in studies of adults and children motivate future developmental studies of autonomic activation during speech and non-speech tasks in typical and atypical speakers.

### Recording Autonomic Signals From Young Children: Methodological Issues

There are additional results from an earlier study from our laboratory (Arnold et al., 2014) in which an experimental design similar to the present study was used to compare normally fluent young adults (18 to 22 years) and school-age children (7 to 9 years) across a similar set of speech/non-speech tasks. The results of this experiment generally accord with our findings that children have higher SNS activation during a Valsalva maneuver, while young adults showed highest levels during a sentence production task. However, it is not possible to directly compare the results of this earlier experiment with the present findings, due to our strategy of using pre-task baseline measures to express relative amplitudes of the autonomic variables. It is well established that there is significant within-individual variability in autonomic measures (Dawson et al., 2007); therefore, experimenters often express task-related amplitudes relative to a baseline condition. Ideally, baseline data is collected immediately prior to performance of each experimental task, so that, for example, adaptation to the experimental environment over time would not affect the measures. This is straightforward to do in adult participants, who are able to rest quietly for a few minutes between experimental tasks (e.g., Weber and Smith, 1990). In Arnold et al. (2014) we used the same procedure, but shortened the inter-task rest periods, because children could not be still and rest for lengthier durations. As a result, some findings from this study were unintuitive and difficult to interpret. Measures of task-related changes from baseline in some cases were lower than the pretrial “rest” baseline intervals, suggesting lower SNS activation during task performance compared to rest. More likely, the pre-task rest interval was not long enough for arousal levels to return to baseline from the previous task and/or simply reflect variability in the child’s behavioral states during the inter-condition rest periods.

This is a significant methodological issue for studies of autonomic activity in children. We began the present investigation employing inter-task rest periods for baseline data, but it became apparent that this was again problematic. We changed our experimental procedures to include a longer rest interval before any experimental tasks were undertaken and controlled for baseline levels of SNS indices in our statistical models. Our results suggest that this procedural change was successful in allowing us to obtain a meaningful comparison of SNS activation across tasks. Namely, the indices of SNS activity (increases in skin conductance and decreases in BPV) follow similar patterns across tasks in both groups of children (Figures 2, 4). As expected, the resting baseline measures were a significant covariate across the SNS dependent variables (Table 1), and SNS variables were highly correlated across tasks (Figure 3). Pilot data made it clear that the high levels of autonomic activity accompanying the forceful blowing task required ample time to resolve. Therefore, we elected not to vary the order of performance of the MAX task, having participants complete it at the end of the experiment. We counterbalanced the order of the JAW, SENT, and PIC tasks across participants. Taken together, these methods yielded results that were not contaminated by task order or adaptation effects.

### Behavioral Measures of Temperament and Communication Attitude

Several studies using parent-report measures have noted temperamental differences between groups of CWS and CWNS, although the constellation of these differences varies and effect sizes reported by Alm (2014) are small. These studies found differences in attention regulation between CWS and CWNS (Karrass et al., 2006), with studies documenting hyperfocus in CWS (Anderson et al., 2003; Eggers et al., 2010). CWS were also less able to regulate their emotions (Karrass et al., 2006) or were vulnerable to increased frustration and anger compared to CWNS (Eggers et al., 2010). Other studies using parent-report measures, however, have not found temperamental differences between CWS and CWNS (Reilly et al., 2013; Kefalianos et al., 2014, 2017). The results of Kefalianos and colleagues are particularly compelling as these measures were sampled longitudinally, beginning prior to the onset of stuttering. We also found similar temperamental profiles in groups of CWS and CWNS; thus, our results do not support the hypothesis that temperamental differences distinguish preschool CWS and CWNS. Our findings, however, do not address the possibility that early temperamental profiles would distinguish children at higher risk for persistent stuttering. Ambrose et al. (2015) noted a significant, albeit small effect, for persisting CWS to have higher negative affectivity compared to both CWS who recovered and CWNS.

Regarding communication attitudes, we found that CWS achieved significantly higher KiddyCAT scores indicating more negative communication attitudes compared to their fluent peers. Interpretation of this finding is complicated by the fact that the mean KiddyCAT scores we calculated for each group were approximately half of the means reported for CWS and CWNS for this measure (Vanryckeghem and Brutten, 2006). Thus, both groups of children self-reported relatively few negative responses on this measure. The KiddyCAT scores we noted do not accord with the scores reported by other studies administering the English version of the KiddyCAT to preschoolers (Vanryckeghem et al., 2005; Clark et al., 2012). Guttormsen et al. (2015) reviewed assessments of communication attitude in children aged 3–18 years and documented negative communication attitudes in CWS beginning in the preschool years with differences between groups of CWS and CWNS becoming more apparent with advancing age. As with the CBQ results, our KiddyCAT findings do not address the role that communication attitudes may play in persistent stuttering.

### Relationships Among Physiological and Behavioral Indices

A logical question is whether indices of physiological arousal levels are related to stuttering severity or to dimensions of temperament as measured by the CBQ. Our analyses of relationships among skin conductance measures and stuttering severity revealed an unexpected, negative correlation between the WSI and SCL for the PIC task (Figure 5). The correlation suggests that children with more severe stuttering have lower levels of task-related SCL. If one examines Figure 5, however, it appears that the correlation is driven by outliers at either end of the scales. The majority of our sample of CWS had WSI scores in the 3–13 range, and the data points within this range of WSI, suggest no systematic relationship with SCL. There is also the problem of the relative undersampling of children with higher WSI scores. Thus, our results are inconclusive on this issue, however, we note that overall our findings suggest no relationship between early childhood stuttering and levels of SNS arousal during speech. We did not compute correlations among skin conductance measures and TOCS or KiddyCat scores, because these score distributions again undersampled the higher end of the two scales. We also found no significant relationships between SNS variables and the CBQ composite scores. Therefore, we conclude that there is no simple mapping between the physiological measures of sympathetic arousal in preschool children during speech and non-speech tasks and any of the behavioral variables.

## Conclusion

Our results do not support the hypothesis we advanced concerning speech-specific increases in SNS arousal in CWS, nor do they support any predictable relationships among behavioral measures of stuttering severity, temperament, and physiological measures of SNS arousal. The finding that preschool children typically do not show the heightened levels of SNS activation during speech tasks has significant implications with regard to sympathetic arousal and speech production over the lifespan. They suggest that the coupling between SNS and speech sensorimotor systems develops with maturation, ultimately resulting in remarkably high levels of sympathetic activation during speech in typically fluent adults and in adults who have continued to stutter. Our study examines CWS at a single time point when these children are stuttering. Within the framework of the Multifactorial Dynamic Pathways Theory (Smith and Weber, 2017), the heterogeneous findings of tonic SCLs in CWS provides an important clue for future studies. It will be important to determine if relatively higher SCLs in some CWS represent a risk marker for persistent stuttering. Furthermore, noting that CWS showed small, but non-significant effect sizes for the measures of SCR frequency and BPV amplitude indicating higher transient SNS arousal compared to CWNS, these measures also warrant investigation as potential predictors of stuttering outcomes. Longitudinal studies are required to map the developmental course of functional linkages among neural systems involved in autonomic control and speech production in CWS as they recover or persist.

## Data Availability Statement

The datasets generated for this study are available on request to the corresponding author.

## Ethics Statement

The studies involving human participants were reviewed and approved by the Purdue University Institutional Review Board. Written informed consent to participate in this study was provided by the participants’ legal guardian/next of kin.

## Author Contributions

BW, AS, and CW conceived and planned the experiments. BW carried out the experiments and supervised the data analysis. SC derived the statistical models and analyzed the data with BW. BW, AS, SC, and CW discussed the results. BW and AS wrote the manuscript with input from SC and CW. All authors reviewed the submitted manuscript.

## Conflict of Interest

The authors declare that the research was conducted in the absence of any commercial or financial relationships that could be construed as a potential conflict of interest.

## References

[B1] Ackerley R. Aimonetti J.-M. Ribot-Ciscar E. (2017). Emotions alter muscle proprioceptive coding of movements in humans. *Sci. Rep.* 7:8465. 10.1038/s41598-017-08721-4 28814736 PMC5559453

[B2] Alm P. A. (2004). Stuttering, emotions, and heart rate during anticipatory anxiety: a critical review. *J. Fluency Disord.* 29 123–133. 10.1016/j.jfludis.2004.02.001 15178128

[B3] Alm P. A. (2014). Stuttering in relation to anxiety, temperament, and personality: review and analysis with focus on causality. *J. Fluency Disord.* 40 5–21. 10.1016/j.jfludis.2014.01.004 24929463

[B4] Ambrose N. G. Yairi E. (1999). Normative disfluency data for early childhood stuttering. *J. Speech Lang. Hear. Res.* 42 895–909. 10.1044/jslhr.4204.895 10450909

[B5] Ambrose N. G. Yairi E. Loucks T. M. Seery C. H. Throneburg R. (2015). Relation of motor, linguistic and temperament factors in epidemiologic subtypes of persistent and recovered stuttering: initial findings. *J. Fluency Disord.* 45 12–26. 10.1016/j.jfludis.2015.05.004 26117417 PMC4546885

[B6] Anderson J. D. Pellowski M. W. Conture E. G. (2005). Childhood stuttering and dissociations across linguistic domains. *J. Fluency Disord.* 30 219–253. 10.1016/j.jfludis.2005.05.006 16045977

[B7] Anderson J. D. Pellowski M. W. Conture E. G. Kelly E. M. (2003). Temperamental characteristics of young children who stutter. *J. Speech Lang. Hear. Res.* 46 1221–1233. 10.1044/1092-4388(2003/095) 14575354 PMC1458369

[B8] Andreassi J. L. (2007). *Psychophysiology: Human Behavior and Physiological Response*, 5 Edn, Mahwah NJ: Lawrence Erlbaum Associates.

[B9] Arbuckle J. (1996). “Full information estimation in the presence of incomplete data,” in *Advanced Structural Equation Modeling: Issues and Techniques*, eds Marcoulides G. A. Schumacker R. E. (Mahwah NJ: Lawrence Erlbaum Associates), 243–277.

[B10] Arnold H. S. MacPherson M. K. Smith A. (2014). Autonomic correlates of speech versus nonspeech tasks in children and adults. *J. Speech Lang. Hear. Res.* 57 1296–1307. 10.1044/2014_JSLHR-S-12-0265 24686989 PMC4307925

[B11] Bates J. E. (1989). “Concepts and measures of temperament,” in *Temperament in Childhood*, eds Kohnstamm G. A. Bates J. E. Rothbart M. K. (Oxford: John Wiley & Sons), 3–26.

[B12] Beuter A. Duda J. L. (1985). Analysis of the arousal/motor performance relationship in children using movement kinematics. *J. Sport Psychol.* 7 229–243. 10.1123/jsp.7.3.229

[B13] Beuter A. Duda J. L. Widule C. J. (1989). The effect of arousal on joint kinematics and kinetics in children. *Res. Q. Exerc. Sport* 60 109–116. 10.1080/02701367.1989.10607425 2489831

[B14] Binder D. A. (1983). On the variances of asymptotically normal estimators from complex surveys. *Int. Stat. Rev.* 51 279–292.

[B15] Boucsein W. (2013). *Electrodermal Activity.* New York, NY: Plenum Press.

[B16] Cardinali D. P. (2018). *Autonomic Nervous System: Basic and Clinical Aspects.* New York, NY: Springer International Publishing.

[B17] Choi D. Conture E. G. Walden T. A. Jones R. M. Kim H. (2016). Emotional diathesis, emotional stress, and childhood stuttering. *J. Speech Lang. Hear. Res.* 59 616–630. 10.1044/2015_JSLHR-S-14-0357 27327187 PMC5280059

[B18] Clark C. E. Conture E. G. Frankel C. B. Walden T. A. (2012). Communicative and psychological dimensions of the KiddyCAT. *J. Commun. Disord.* 45 223–234. 10.1016/j.jcomdis.2012.01.002 22333753 PMC3334450

[B19] Critchley H. D. (2005). Neural mechanisms of autonomic, affective, and cognitive integration. *J. Comp. Neurol.* 493 154–166. 10.1002/cne.20749 16254997

[B20] Critchley H. D. (2009). Psychophysiology of neural, cognitive and affective integration: fMRI and autonomic indicants. *Int. J. Psychophysiol.* 73 88–94. 10.1016/j.ijpsycho.2009.01.012 19414044 PMC2722714

[B21] Dawson M. E. Schell A. M. Filion D. L. (2007). “The electrodermal system,” in *Handbook of Psychophysiology*, 3rd Edn, eds Cacioppo J. T. Tassinary L. G. Berntson G. G. (New York, NY: Cambridge University Press), 159–181.

[B22] Eggers K. De Nil L. F. Van den Bergh B. R. H. (2010). Temperament dimensions in stuttering and typically developing children. *J. Fluency Disord.* 35 355–372. 10.1016/j.jfludis.2010.10.004 21130269

[B23] Ehrler D. J. McGhee R. L. (2008). *PTONI: Primary Test of Nonverbal Intelligence.* Austin, TX: Pro-Ed.

[B24] Enders C. K. Bandalos D. L. (2001). The relative performance of full information maximum likelihood estimation for missing data in structural equation models. *Struct. Equ. Model. Multidiscip. J.* 8 430–457. 10.1207/s15328007sem0803_5

[B25] Frayne E. Coulson S. Adams R. Croxson G. Waddington G. (2016). Proprioceptive ability at the lips and jaw measured using the same psychophysical discrimination task. *Exp. Brain Res.* 234 1679–1687. 10.1007/s00221-016-4573-0 26860522

[B26] Fredrikson M. Furmark T. Olsson M. T. Fischer H. Andersson J. Långström B. (1998). Functional neuroanatomical correlates of electrodermal activity: a positron emission tomographic study. *Psychophysiology* 35 179–185. 10.1017/s0048577298001796 9529944

[B27] Gillam R. Logan K. Pearson N. (2009). *Test of Childhood Stuttering.* Austin: Pro-Ed.

[B28] Grossman P. Taylor E. W. (2007). Toward understanding respiratory sinus arrhythmia: relations to cardiac vagal tone, evolution and biobehavioral functions. *Biol. Psychol.* 74 263–285. 10.1016/j.biopsycho.2005.11.014 17081672

[B29] Guttormsen L. S. Kefalianos E. Naess K.-A. B. (2015). Communication attitudes in children who stutter: a meta-analytic review. *J. Fluency Disord.* 46 1–14. 10.1016/j.jfludis.2015.08.001 26365773

[B30] Hollingshead A. (1975). *A Four-Factor Index of Social Status.* New Haven, CT: Yale University.

[B31] Iani C. Gopher D. Lavie P. (2004). Effects of task difficulty and invested mental effort on peripheral vasoconstriction. *Psychophysiology* 41 789–798. 10.1111/j.1469-8986.2004.00200.x 15318885

[B32] Jänig W. (2006). *The Integrative Action of the Autonomic Nervous System.* Cambridge: Cambridge University Press.

[B33] Jones R. M. Buhr A. P. Conture E. G. Tumanova V. Walden T. A. Porges S. W. (2014b). Autonomic nervous system activity of preschool-age children who stutter. *J. Fluency Disord.* 41 12–31. 10.1016/j.jfludis.2014.06.002 25087166 PMC4150817

[B34] Jones R. Choi D. Conture E. Walden T. (2014a). Temperament, emotion, and childhood stuttering. *Semin. Speech Lang.* 35 114–131. 10.1055/s-0034-1371755 24782274 PMC4317269

[B35] Karrass J. Walden T. A. Conture E. G. Graham C. G. Arnold H. S. Hartfield K. N. (2006). Relation of emotional reactivity and regulation to childhood stuttering. *J. Commun. Disord.* 39 402–423. 10.1016/j.jcomdis.2005.12.004 16488427 PMC1630450

[B36] Kefalianos E. Onslow M. Ukoumunne O. Block S. Reilly S. (2014). Stuttering, temperament, and anxiety: data from a community cohort ages 2-4 years. *J. Speech Lang. Hear. Res.* 57 1314–1322. 10.1044/2014_JSLHR-S-13-0069 24687124

[B37] Kefalianos E. Onslow M. Ukoumunne O. C. Block S. Reilly S. (2017). Temperament and early stuttering development: cross-sectional findings from a community cohort. *J. Speech Lang. Hear. Res.* 60 772–784. 10.1044/2016_JSLHR-S-15-0196 28359081

[B38] Kleinow J. Smith A. (2006). Potential interactions among linguistic, autonomic, and motor factors in speech. *Dev. Psychobiol.* 48 275–287. 10.1002/dev.20141 16617462

[B39] Kreibig S. D. (2010). Autonomic nervous system activity in emotion: a review. *Biol. Psychol.* 84 394–421. 10.1016/j.biopsycho.2010.03.010 20371374

[B40] Kreidler K. Hampton Wray A. Usler E. Weber C. (2017). Neural indices of semantic processing in early childhood distinguish eventual stuttering persistence and recovery. *J. Speech Lang. Hear. Res.* 60 3118–3134. 10.1044/2017_JSLHR-S-17-0081 29098269 PMC5945075

[B41] Lim C. L. Seto-Poon M. Clouston P. D. Morris J. G. L. (2003). Sudomotor nerve conduction velocity and central processing time of the skin conductance response. *Clin. Neurophysiol.* 114 2172–2180. 10.1016/s1388-2457(03)00204-9 14580616

[B42] MacPherson M. K. Abur D. Stepp C. E. (2017). Acoustic measures of voice and physiologic measures of autonomic arousal during speech as a function of cognitive load. *J. Voice* 31 .e1–.e504. 10.1016/j.jvoice.2016.10.021 27939119 PMC6081741

[B43] MacPherson M. K. Smith A. (2013). Influences of sentence length and syntactic complexity on the speech motor control of children who stutter. *J. Speech Lang. Hear. Res.* 56 89–102. 10.1044/1092-4388(2012/11-0184) 22490621 PMC3918903

[B44] Mendes W. B. (2009). “Assessing the autonomic nervous system,” in *Methods in Social Neuroscience*, eds Harmon-Jones E. Beer J. S. (New York, NY: Guilford Press), 118–147.

[B45] Miller J. Iglesias A. (2006). *Systematic Analysis of Language Transcripts (SALT) (Version 9).* Madison, WI: University of Wisconsin-Madison.

[B46] Nagai Y. Critchley H. D. Featherstone E. Trimble M. R. Dolan R. J. (2004). Activity in ventromedial prefrontal cortex covaries with sympathetic skin conductance level: a physiological account of a “default mode” of brain function. *NeuroImage* 22 243–251. 10.1016/j.neuroimage.2004.01.019 15110014

[B47] Noteboom J. T. Fleshner M. Enoka R. M. (2001). Activation of the arousal response can impair performance on a simple motor task. *J. Appl. Physiol.* 91 821–831. 10.1152/jappl.2001.91.2.821 11457799

[B48] Oldfield R. C. (1971). The assessment and analysis of handedness: the Edinburgh inventory. *Neuropsychologia* 9 97–113. 10.1016/0028-3932(71)90067-4 5146491

[B49] Oppenheimer S. M. Gelb A. Girvin J. P. Hachinski V. C. (1992). Cardiovascular effects of human insular cortex stimulation. *Neurology* 42 1727–1732. 1513461 10.1212/wnl.42.9.1727

[B50] Passatore M. Roatta S. (2006). Influence of sympathetic nervous system on sensorimotor function: whiplash associated disorders (WAD) as a model. *Eur. J. Appl. Physiol.* 98 423–449. 10.1007/s00421-006-0312-8 17036216

[B51] Pereira M. G. de Oliveira L. Erthal F. S. Joffily M. Mocaiber I. F. Volchan E. (2010). Emotion affects action: midcingulate cortex as a pivotal node of interaction between negative emotion and motor signals. *Cogn. Affect. Behav. Neurosci.* 10 94–106. 10.3758/CABN.10.1.94 20233958 PMC2875262

[B52] Peters H. F. Hulstijn W. (1984). Stuttering and anxiety: the difference between stutterers and nonstutterers in verbal apprehension and physiologic arousal during the anticipation of speech and non-speech tasks. *J. Fluency Disord.* 9 67–84. 10.1016/0094-730x(84)90008-1

[B53] Putnam S. P. Rothbart M. K. (2006). Development of short and very short forms of the Children’s Behavior Questionnaire. *J. Pers. Assess.* 87 102–112. 10.1207/s15327752jpa8701_09 16856791

[B54] Radovanovic D. Peikert K. Lindström M. Domellöf F. P. (2015). Sympathetic innervation of human muscle spindles. *J. Anat.* 226 542–548. 10.1111/joa.12309 25994126 PMC4450958

[B55] Reilly S. Onslow M. Packman A. Cini E. Conway L. Ukoumunne O. C. (2013). Natural history of stuttering to 4 years of age: a prospective community-based study. *Pediatrics* 132 460–467. 10.1542/peds.2012-3067 23979093

[B56] Roatta S. Windhorst U. Ljubisavljevic M. Johansson H. Passatore M. (2002). Sympathetic modulation of muscle spindle afferent sensitivity to stretch in rabbit jaw closing muscles. *J. Physiol.* 540 237–248. 10.1113/jphysiol.2001.014316 11927683 PMC2290222

[B57] Schopler E. Van Bourgondien M. E. Wellman G. J. Love S. R. (2010). *CARS-2: Childhood Autism Rating Scale Second Edition.* Los Angles, CA: Western Psychological Services.

[B58] Silverman S. Ratner N. B. (2002). Measuring lexical diversity in children who stutter: application of vocd. *J. Fluency Disord.* 27 289–304. 10.1016/s0094-730x(02)00162-6 12506447

[B59] Simpson J. R. Drevets W. C. Snyder A. Z. Gusnard D. A. Raichle M. E. (2001). Emotion-induced changes in human medial prefrontal cortex: II. During anticipatory anxiety. *Proc. Natl. Acad. Sci. U.S.A.* 98 688–693. 10.1073/pnas.98.2.688 11209066 PMC14649

[B60] Smith A. (1992). The control of orofacial movements in speech. *Crit. Rev. Oral Biol. Med.* 3 233–267. 10.1177/10454411920030030401 1571473

[B61] Smith A. Weber C. (2017). How stuttering develops: the multifactorial dynamic pathways theory. *J. Speech Lang. Hear. Res.* 60 2483–2505. 10.1044/2017_JSLHR-S-16-0343 28837728 PMC5831617

[B62] Spencer C. Weber-Fox C. (2014). Preschool speech articulation and nonword repetition abilities may help predict eventual recovery or persistence of stuttering. *J. Fluency Disord.* 41 32–46. 10.1016/j.jfludis.2014.06.001 25173455 PMC4150085

[B63] Usler E. Smith A. Weber C. (2017). A lag in speech motor coordination during sentence production is associated with stuttering persistence in young children. *J. Speech Lang. Hear. Res.* 60 51–61. 10.1044/2016_JSLHR-S-15-0367 28056137 PMC5533560

[B64] Vanryckeghem M. Brutten G. J. (2006). *KiddyCAT Communication Attitude Test for Preschool and Kindergarten Children Who Stutter.* San Diego, CA: Plural Publishing.

[B65] Vanryckeghem M. Brutten G. J. Hernandez L. M. (2005). A comparative investigation of the speech-associated attitude of preschool and kindergarten children who do and do not stutter. *J. Fluency Disord.* 30 307–318. 10.1016/j.jfludis.2005.09.003 16246410

[B66] Venables P. H. Christie M. J. (1980). *Electrodermal Activity. Techniques in Psychophysiology.* Chichester: Wiley.

[B67] Vickers J. N. Williams A. M. (2007). Performing under pressure: the effects of physiological arousal, cognitive anxiety, and gaze control in biathlon. *J. Mot. Behav.* 39 381–394. 10.3200/jmbr.39.5.381-394 17827115

[B68] Vissing S. F. Scherrer U. Victor R. G. (1991). Stimulation of skin sympathetic nerve discharge by central command. Differential control of sympathetic outflow to skin and skeletal muscle during static exercise. *Circ. Res.* 69 228–238. 10.1161/01.res.69.1.228 2054936

[B69] Vissing S. F. Secher N. H. Victor R. G. (1997). Mechanisms of cutaneous vasoconstriction during upright posture. *Acta Physiol. Scand.* 159 131–138. 10.1046/j.1365-201x.1997.573344000.x 9055940

[B70] Walden T. A. Frankel C. B. Buhr A. P. Johnson K. N. Conture E. G. Karrass J. M. (2012). Dual diathesis-stressor model of emotional and linguistic contributions to developmental stuttering. *J. Abnorm. Child Psychol.* 40 633–644. 10.1007/s10802-011-9581-8 22016200 PMC3740566

[B71] Walsh B. Mettel K. M. Smith A. (2015). Speech motor planning and execution deficits in early childhood stuttering. *J. Neurodev. Disord.* 7:27. 10.1186/s11689-015-9123-8 26300988 PMC4545974

[B72] Walsh B. Usler E. (in press). Physiological correlates of fluent and stuttered speech production in preschoolers who stutter. *J. Speech Lang. Hear. Res.* 10.1044/2019_JSLHR-S-19-0018 PMC7201324 31805242

[B73] Weber C. M. Smith A. (1990). Autonomic correlates of stuttering and speech assessed in a range of experimental tasks. *J. Speech Hear. Res.* 33 690–706. 10.1044/jshr.3304.690 2273884

[B74] Weber-Fox C. Hampton Wray A. Arnold H. (2013). Early childhood stuttering and electrophysiological indices of language processing. *J. Fluency Disord.* 38 206–221. 10.1016/j.jfludis.2013.01.001 23773672 PMC3687214

[B75] Williams L. M. Phillips M. L. Brammer M. J. Skerrett D. Lagopoulos J. Rennie C. (2001). Arousal dissociates amygdala and hippocampal fear responses: evidence from simultaneous fMRI and skin conductance recording. *NeuroImage* 14 1070–1079. 10.1006/nimg.2001.0904 11697938

[B76] Yoshie M. Kudo K. Ohtsuki T. (2009). Motor/autonomic stress responses in a competitive piano performance. *Ann. N. Y. Acad. Sci.* 1169 368–371. 10.1111/j.1749-6632.2009.04786.x 19673810

[B77] Yoshie M. Nagai Y. Critchley H. D. Harrison N. A. (2016). Why I tense up when you watch me: inferior parietal cortex mediates an audience’s influence on motor performance. *Sci. Rep.* 6:19305 10.1038/srep19305 PMC4726313 26787326

[B78] Zengin-Bolatkale H. Conture E. G. Walden T. A. (2015). Sympathetic arousal of young children who stutter during a stressful picture naming task. *J. Fluency Disord.* 46 24–40. 10.1016/j.jfludis.2015.07.005 26296616 PMC4877440

[B79] Zengin-Bolatkale H. Conture E. G. Walden T. A. Jones R. M. (2018). Sympathetic arousal as a marker of chronicity in childhood stuttering. *Dev. Neuropsychol.* 43 135–151. 10.1080/87565641.2018.1432621 29412003 PMC5826607

[B80] Zimmermann G. (1980). Stuttering: a disorder of movement. *J. Speech Lang. Hear. Res.* 23 122–136. 10.1044/jshr.2301.122 6777605

